# Editorial: Mental health of disadvantaged children

**DOI:** 10.3389/fpsyt.2022.1130118

**Published:** 2023-01-09

**Authors:** Junfeng Zhao, Huang Gu, Boliang Guo, Xiaoming Li

**Affiliations:** ^1^Institute of Behavior and Psychology, School of Psychology, Henan University, Kaifeng, China; ^2^Medical Statistics, School of Medicine, University of Nottingham, Nottingham, United Kingdom; ^3^Department of Health Promotion, Education, and Behavior, University of South Carolina, Columbia, SC, United States

**Keywords:** disadvantage children, mental health, intervention strategy, developmental psychology, resilience

There is a significant amount of research on the mental health of disadvantaged children worldwide. According to the literature, disadvantaged children include children orphaned by HIV/AIDS, children left behind by their migratory parents (“left-behind children”), immigrant children, children with learning disabilities, and children with physical challenges (e.g., deaf children). Further, disadvantaged children face numerous challenges during childhood, such as poverty, disrupted school attendance, lack of parental care, and stigmatization. These negative events can cause significant stress for children and have long-term negative impacts on their mental health. Although previous literature has discussed the development of mental health among different disadvantaged groups, there is limited research that comprehensively examines the developmental trends and intervention strategies for disadvantaged children.

The current Research Topic explores issues among various disadvantaged groups by using psychological research techniques to learn from the collective work across different regions, countries, cultures, and ages. This Research Topic has produced numerous broad representative findings from a total of 15 academic papers that involved 61 authors from 19 schools in four countries: China, United States (US), Zambia, and South Africa. The studies employed a variety of research methodologies including questionnaire surveys, laboratory experiments, literature reviews, and meta-analyses.

Compared to other groups, disadvantaged children have less power, fewer rights, and less access to resources and conditions for daily life, education and future career development, which causes many disorders in their physical and mental development, cognitive function, and academic performance. Furthermore, disadvantaged children are diverse and large in number, which brings the detrimental realities disadvantaged children face to the forefront of larger public conversations. As a result, researchers in various fields have conducted a series of studies on the physical and mental development of disadvantaged children and have achieved many empirical findings and constructive conclusions. Additionally, governments and civil society in general have introduced numerous policies and programs to promote the healthy physical and psychological development of disadvantaged children.

The health and development of disadvantaged children includes two main themes: physical health development and promotion, and educational and psychological development (including mental health, cognitive, and social development). The vast majority of studies focus on mental health and cognitive development, with far less research focused on social development. Researchers exploring mental health development have focused on the effects of protective and risk factors on mental health. Numerous studies have shown that resilience and social support could significantly predict the mental health of disadvantaged children. For example, Du et al. (1) reported that meaning in life and resilience could significantly predict psychological outcomes (i.e., loneliness and depression) among children affected by parental HIV. More importantly, resilience moderated the relationship between meaning in life and depression. Jiang et al. (2) recently investigated the effects of a resilience-based intervention on the mental health of children affected by parental HIV and found that the intervention yielded significant improvements in positive coping and had benefits on individual depression, loneliness, and anxiety.

Mebrahtu et al. (3) focused on the potential risk and protective factors on children's cognitive development and found that increased maternal education levels protected against lower child developmental scores while increasing child age was a risk factor for lower developmental scores. Zhang Y. et al. showed that perceived social support and trust could positively predict social wellbeing among youths affected by parental HIV/AIDS, implying trust appears to be the most proximate protective factor for social wellbeing. In addition, studies found that stigma had negative effects on the development of psychological wellbeing. For instance, Chi et al. (4) discovered that perceived stigma negatively predicted resilience among children affected by HIV, implying that perceived stigma also affected mental health.

In a study of the effect of stigma on social wellbeing in children affected by AIDS, enacted stigma moderated the relationship between perceived social support and social trust. The effect of enacted social support on social trust was greater for children with low-perceived stigma than for those with high-perceived stigma. Meanwhile, perceived stigma moderated the relationship between social trust and social wellbeing with the positive predictive effect of social trust on social wellbeing being stronger among those adolescents who perceived more stigma (Zhang F. et al.). These studies show that protective factors such as psychological resilience and perceived social support can have a positive effect on mental health, alternatively, risk factors such as stigma have a negative effect on mental health.

Research on cognitive development has explored the cognitive processing of learning. In a study exploring the effects of executive function of students with mathematics or reading disabilities on prospective memory (PM), researchers found that students with mathematics or reading disabilities suffer from the deficits of prospective memory and executive function. Moreover, executive function significantly predicted PM performance (5). In Zhao et al.'s (6) study of children affected by AIDS, children with early adversity suffered from working memory deficits. A mechanistic study of PM in children with learning disability (LD) using event-related potentials found that children with LD have worse PM performance which manifests as a selective deficit in PM cues detection, rather than the absence of PM intention retrieval (Ji et al.).

Some intervention efforts have been made to promote the health and psychosocial development of disadvantaged children. Harrison et al. (7) reported a multilevel psychosocial promotion intervention (child, caregiver, and community) conducted by a Sino-US international collaborative team among children affected by parental HIV/AIDS. The team adopted a multidisciplinary perspective of education, psychology, sociology and physiology to explore the health development and promotion of these children. The study reported that the academic performance, school satisfaction and interest in learning of children who participated in the intervention steadily increased over an 18-month period (7). The study also found that psychological resilience significantly predicted individual support-seeking and positive emotion. The intervention group showed greater growth in psychological resilience, support-seeking and positive emotion than the control group at 6-month follow-up. The intervention group also displayed significantly more post-traumatic growth than the control group at 12-month follow-up.

Research for the health and development of disadvantaged children commonly utilize questionnaire surveys for assessment. While this popular method is more subjective by nature, some studies in this issue demonstrate the utility of objective biological indicators in the exploration of mental health and development of disadvantaged children. For example, diurnal cortisol was used to study the negative family expressiveness and internalizing problems among left-behind adolescents (Li et al.). Multiple papers in this issue have used the event-related potential (ERP) technique to explore the brain mechanisms of psychological development in disadvantaged children. Gu et al. found that the microstate and functional connectivity has altered in children orphaned by HIV and early life stress (ELS) would alter the structure and function of the brain and increase the risk of psychiatric disorders. Ji et al. applied ERP technique to explore the event-based prospective memory (EBPM) in 21 children with LD and 20 non-LD children and found that the poor performance of LD children on PM tasks may result from deficits in PM cues detection. Wan et al. used ERP technique to explore the effect of social exclusion on trust in 31 AIDS orphans (32 age and development status matched controls) and found that orphans might have formed some self-protective mechanisms to prevent trauma from the negative feedback of others.

Currently, an emerging research theme in the study of disadvantaged children is the exploration of developmental coordination disorders. This growing research field has expanded to advanced cognitive processing processes such as attention, working memory and executive function (8–11). The results show that adolescents with developmental coordination disorder have common cognitive processing defects. Subsequently, the researchers focused on the characteristics of the neural mechanism of visual spatial attention of adolescents with developmental coordination disorder and carried out a series of explorations (12). The results showed that adolescents with developmental coordination disorder had visual spatial attention deficits to varying degrees. In future research, PET, ERP, FMRI and other EEG technologies will be used to further explore the neural mechanism of developmental coordination disorder.

The current Research Topic brings together theoretical and empirical advancements that address the mental health of disadvantaged children. Still, the studies reported here may not be comprehensive and conclusions need to be considered in the light of various conceptual and methodological limitations described within the articles. This issue is also subject to some geographic limitation as most of the studies were conducted in China. However, we hope that this Research Topic will serve as a call for more studies worldwide to build a more comprehensive evidence base (13). We hope that the global community will commit to further exploration of innovative and diligent research for the mental disorders and neurological changes that result from adverse environments, as well as the effective intervention strategies and policies to improve the mental health of disadvantaged children around the globe.

## Author contributions

XL and JZ conceptualized the paper. JZ and HG developed the first draft. XL and BG provided critical edits and revisions. All authors contributed to the article and approved the submitted version.
